# Aminoglycosides enhance meropenem/vaborbactam activity against KPC-producing *Klebsiella pneumoniae* in the hollow fiber infection model

**DOI:** 10.1128/aac.01365-25

**Published:** 2026-02-19

**Authors:** Nidhi Singh, Christin M. L. Jogan, Yanan Zang, Xindi Shan, Yinzhi Lang, Arunkumar Karunanidhi, Jackson V. Watkins, Brooke N. Curry, Pranita D. Tamma, Sophie H. Nozick, Egon A. Ozer, Alan R. Hauser, Jürgen B. Bulitta, Zackery P. Bulman

**Affiliations:** 1 Department of Pharmacy Practice, Retzky College of Pharmacy, University of Illinois Chicago 14681, Chicago, Illinois, USA; 2 Department of Pharmacotherapy and Translational Research, College of Pharmacy, University of Florida 15505, Orlando, Florida, USA; 3 Department of Pharmacy and Pharmaceutical Sciences, St. Jude Children's Research Hospital 5417, Memphis, Tennessee, USA; 4 Department of Pediatrics, Division of Infectious Diseases, Johns Hopkins University School of Medicine 1500 , Baltimore, Maryland, USA; 5 Department of Microbiology-Immunology, Northwestern University Feinberg School of Medicine 12244, Chicago, Illinois, USA; 6 Division of Infectious Diseases, Department of Medicine, Northwestern University Medical School 3270, Chicago, Illinois, USA; Providence Portland Medical Center, Portland, Oregon, USA

**Keywords:** meropenem/vaborbactam, combinations, pharmacokinetics/pharmacodynamics, hollow fiber infection model, CRE, mutation frequency

## Abstract

Meropenem/vaborbactam is a preferred treatment option for KPC-producing *Klebsiella pneumoniae* (KPC-Kp) infections, but clinical cure rates remain suboptimal when it is used alone. This study aimed to assess the pharmacodynamic activity of meropenem/vaborbactam alone and in combination with an aminoglycoside in the hollow fiber infection model (HFIM). The HFIM was used to simulate meropenem/vaborbactam and aminoglycoside pharmacokinetic profiles that approximated antibiotic exposures in the plasma and lung epithelial lining fluid (ELF) following human doses against four meropenem/vaborbactam-susceptible KPC-Kp isolates. Two isolates had lower meropenem/vaborbactam MICs (0.125/8 to 0.25/8 mg/L; NU-CRE105 and NU-CRE244), and two had higher MICs (2/8 mg/L; AR-1049 and AR-1054). Against NU-CRE105 and NU-CRE244, meropenem/vaborbactam was bactericidal and caused >5.7 log_10_ CFU/mL reductions by 48 h. Antibiotic exposures mimicking those in plasma and ELF yielded similar bacterial killing. Despite the robust activity of meropenem/vaborbactam alone, combinations with an aminoglycoside were synergistic, providing ≥2 log_10_ CFU/mL better killing of NU-CRE105 and NU-CRE244 than either monotherapy for ~40% of the experiment. Against AR-1049 and AR-1054, meropenem/vaborbactam monotherapy mimicking ELF exposures generated 2.9–5.2 log_10_ CFU/mL reductions at 48 h. However, meropenem/vaborbactam resistance emerged by 168 h. Combinations with an aminoglycoside displayed 4.7–7.5 log_10_ CFU/mL greater killing than either monotherapy at 168 h and repressed meropenem/vaborbactam resistance. Meropenem/vaborbactam remains an important agent against KPC-Kp. However, pneumonia caused by KPC-Kp isolates with MICs near the susceptibility breakpoint (≤4/8 mg/L) may reduce the pharmacodynamic activity of meropenem/vaborbactam and permit resistance to emerge. Aminoglycosides represent a promising adjunct to meropenem/vaborbactam for select KPC-Kp isolates, owing to their capacity to enhance bacterial killing and suppress resistance.

## INTRODUCTION

The emergence of carbapenem-resistant *Enterobacterales* (CRE) poses a significant threat to patients, given their resistance to multiple antibiotic classes and their association with high mortality rates (1). Among CRE, carbapenem-resistant *Klebsiella pneumoniae*, including those producing the *K. pneumoniae* carbapenemase (KPC-Kp), were listed as the top priority pathogen by the World Health Organization’s 2024 Bacterial Priority Pathogens List (2). For invasive KPC-Kp infections, β-lactam/β-lactamase inhibitor combinations with activity against KPC enzymes, such as meropenem/vaborbactam, are considered preferred treatment options (3).

Vaborbactam is a boronic acid-based β-lactamase inhibitor that has potent activity against the KPC enzyme and is combined with meropenem for clinical use (4). When used to treat infections caused by CRE, 30-day mortality rates following treatment with meropenem/vaborbactam were 9%–16%, which represents an improvement over historically prescribed therapies such as the polymyxins (5–8). However, higher mortality rates (20%–32%) have been observed in patients with more severe illness or comorbidities (9, 10). Furthermore, clinical cure or success with meropenem/vaborbactam occur for only ~60%–70% of patients (5–8), leaving 30%–40% with persistent signs/symptoms of infection or recurrent infections. Thus, certain subsets of patients, such as those who are critically ill, may benefit from enhanced therapeutic options such as an optimized dosage regimen with a second antibiotic. Although a second gram-negative active agent is added to meropenem/vaborbactam therapy in up to 37% of patients in real-world scenarios, these combination regimens have not been rigorously tested or rationally optimized. Strategically combining meropenem/vaborbactam with another antibiotic, such as an aminoglycoside, may enhance bacterial killing and resistance suppression. This study aimed to characterize the pharmacodynamic activity of aminoglycoside and meropenem/vaborbactam combinations against KPC-Kp isolates exhibiting a range of meropenem/vaborbactam MICs.

## MATERIALS AND METHODS

### Antibiotics and MICs

Antibiotics used in this study included meropenem (Sigma-Aldrich), vaborbactam (MedChem Express), meropenem/vaborbactam (Vabomere; clinical-grade powder), gentamicin (Sigma-Aldrich), and plazomicin (ZEMDRI; clinical-grade powder). Meropenem and vaborbactam analytical-grade powder were purchased for MIC testing, while meropenem/vaborbactam powder was used for all other experiments. MICs were determined in triplicate using broth microdilution (BMD) in CAMHB at 37°C, and modal MICs were reported. Drug stocks were freshly prepared on the day of each experiment with quality control strains following Clinical and Laboratory Standards Institute (CLSI) M100 recommendations (11). Meropenem/vaborbactam MICs ≤4/8 µg/mL were defined as susceptible.

### Mutation frequency assay

Sixteen KPC-Kp isolates were used for initial meropenem/vaborbactam MIC and mutation frequency testing, as previously described (Table 1) (12). Isolates 39351 and 46561 were obtained from global *K. pneumoniae* collections (13, 14), NU-CRE isolates originated from Northwestern Memorial Hospital (Chicago, IL, USA) (15), and the AR isolates were obtained from the CDC AR Isolate Bank. A culture of ~10^9^ CFU/mL of each KPC-Kp isolate was prepared by resuspending colonies in CAMHB, and 50 µL aliquots were then transferred to either agar plates embedded with meropenem/vaborbactam (8/8 mg/L) or a control plate without antibiotic. Plates were incubated at 37°C for 24 h, and viable colonies were counted using an automated colony counter (ProtoCOL 3; Synbiosis). The mutation frequency was determined by dividing the viable counts on meropenem/vaborbactam-containing agar by those on antibiotic-free agar plates.

**TABLE 1:** Meropenem/vaborbactam mutation frequencies on agar containing meropenem/vaborbactam 8/8 mg/L for KPC-Kp isolates with varying MICs^*a*^

Strain	MIC (mg/L)	Meropenem/vaborbactam resistance frequency
39351	0.03/8	<1.58 × 10^−9^
46561	0.03/8	<1.36 × 10^−9^
NU-CRE002	0.03/8	<1.36 × 10^−9^
NU-CRE027	0.03/8	<1.54 × 10^−9^
NU-CRE078	0.03/8	<1.64 × 10^−9^
NU-CRE243	0.03/8	<1.78 × 10^−9^
NU-CRE251	0.03/8	<1.66 × 10^−9^
NU-CRE050	0.125/8	<1.80 × 10^−9^
NU-CRE105	0.125/8	<2.38 × 10^−9^
NU-CRE244	0.25/8	<2.04 × 10^−9^
NU-CRE266	0.5/8	<1.18 × 10^−9^
NU-CRE165	1/8	<2.94 × 10^−9^
AR-1045	2/8	<1.76 × 10^−9^
AR-1049	2/8	**2.65 × 10^−7^**
AR-1052	2/8	<1.54 × 10^−9^
AR-1054	2/8	**3.19 × 10^−7^**

^*a*^ Bold, isolates with resistance frequencies >1 × 10^−9^.

### Time-kill assays

Time-kill assays were performed in duplicate to evaluate the activity of meropenem/vaborbactam against four KPC-Kp isolates, AR-1045, AR-1049, AR-1052, and AR-1054, which had the same MIC but differing mutation frequencies. Meropenem/vaborbactam was tested at clinically relevant concentrations of 4/4, 8/8, 16/16, and 25/25 mg/L against bacterial inocula of ~10^8^ CFU/mL growing in CAMHB at 37°C (16). Viable counts were quantified at 0, 2, 4, 6, 24, and 48 h by plating appropriately diluted bacterial samples on Mueller-Hinton agar (MHA) plates and counting colonies after 1 day of incubation.

### Hollow fiber infection model

Four of the KPC-Kp isolates NU-CRE105, NU-CRE-244, AR-1049, and AR-1054 were tested in the hollow fiber infection model (HFIM). Gentamicin MICs were 0.5 mg/L for NU-CRE105 and NU-CRE244, >128 mg/L for AR-1049, and 1 mg/L for AR-1054. Plazomicin MIC testing was conducted for AR-1049 only, yielding a value of 0.5 mg/L. The HFIM was used to quantify the pharmacodynamic activity of meropenem/vaborbactam alone and in combination with gentamicin or plazomicin, as previously described (17, 18). Prior to the experiment, the C8008 hollow fiber cartridges (FiberCell) were conditioned by flushing 3–4 L of CAMHB through each cartridge. Bacteria were inoculated at ~10^8^ CFU/mL in the cartridges and allowed to grow for ~1 h at 37°C to reach the exponential growth phase before antibiotic dosing began. The HFIM experiments were conducted in two phases. First, shorter-duration runs were used to evaluate site-specific pharmacodynamic activity of the antibiotics by using pharmacokinetic (PK) profiles that simulated plasma and lung epithelial lining fluid (ELF) exposures in humans. Isolates NU-CRE105 and NU-CRE-244 had meropenem/vaborbactam MICs of 0.125/8 and 0.25/8 mg/L, respectively, and were tested for these shorter 48 h duration experiments. Second, a series of longer-duration runs were used to test for the emergence of resistance. Isolates AR-1049 and AR-1054 (meropenem/vaborbactam MICs of 2/8 mg/L) were tested over 168 h to quantify resistance emergence or suppression.

Serial viable counts were determined at pre-specified time points during each experiment by removing a sample of the bacteria from the cartridge, serially diluting, and then plating on MHA. Samples were concurrently plated on antibiotic-containing agar for the 168 h HFIM experiments to characterize resistance emergence. Viable bacteria were enumerated using an automated colony counter after 24 h of incubation on drug-free MHA plates and after 48 h for drug-containing MHA plates. Bactericidal activity was defined as a ≥3-log_10_ CFU/mL reduction by the antibiotic compared to growth at baseline (i.e., 0 h). Synergy was defined as a ≥2-log_10_ CFU/mL reduction by the combination compared to the more active agent in monotherapy (19).

The antibiotics were injected into the central reservoir using automated dosing syringe pumps (New Era Pump Systems Inc.). Predicted PK parameters tested in the HFIM are shown in Table 2. Meropenem/vaborbactam PK imitated concentrations in human plasma and the ELF following a dose of 2 g/2 g q8h, infused over 3 h (16, 20, 21). Similarly, gentamicin PK mimicked concentration profiles in plasma and the ELF following a dose of 5 mg/kg q24h and a 30 min infusion (22–24). An ELF concentration profile following plazomicin doses of 15 mg/kg q24h with an infusion over 30 min was mimicked (22, 25). No plasma plazomicin profile was tested, since this antibiotic was only included in the longer-duration experiments.

**TABLE 2:** Simulated and observed PK profiles in the HFIM^*a*^

Antibiotic	Plasma or ELF	*f*AUC_24_ (mg*h/L)		*fC*_max_ (mg/L)		CL (L/h)		*T*_1/2_ (h)	
		Simulated	Observed	Simulated	Observed	Simulated	Observed	Simulated	Observed
Meropenem^*b*^	Plasma	517.6	471.1	50.2	39.6	0.12	0.12	1.0	1.6
	ELF	258.8	251.8	25.1	20.6	0.12	0.11	1.0	1.7
Vaborbactam^*b*^	Plasma	517.6	524.9	50.2	42.7	0.12	0.11	1.0	1.6
	ELF	258.8	315.2	25.1	24.6	0.12	0.092	1.0	1.7
Gentamicin^*c*^	Plasma	25.9	27.9	15.0	–^*e*^	0.12	0.11	1.0	2.3
	ELF	8.6	11.9	5.0	4.9	0.12	0.087	1.0	1.3
Plazomicin^*d*^	ELF	8.6	8.4	5.0	4.0	0.12	0.12	1.0	1.3

^*a*^ Antibiotic concentrations were dosed to approximate exposures for each antibiotic in plasma and ELF. Data for observed PKs were based on a noncompartmental analysis of the actual antibiotic concentrations in the HFIM central reservoir following the 24 h dose, which were quantified using ultra-performance liquid chromatography-tandem mass spectrometry (UPLC-MS/MS). Observed data were obtained in drug-only experimental runs, except for the plasma data for gentamicin, which was based on samples collected during an experiment with bacteria.

^*b*^ Simulating a human dose of 4 g (2 g/2 g) every 8 h, infused over 3 h.

^*c*^ Simulating a human dose of 5 mg/kg every 24 h, infused over 0.5 h.

^*d*^ Simulating a human dose of 15 mg/kg every 24 h, infused over 0.5 h.

^*e*^ Samples were not collected until 0.5 h after the infusion ended.

### Whole-genome sequencing

Four bacterial samples obtained at 168 h in the HFIM experiments (AR-1049 growth control; AR-1049 meropenem/vaborbactam; AR-1054 growth control; AR-1054 meropenem/vaborbactam) were selected for short- and long-read whole-genome sequencing (WGS). The AR-1049 and AR-1054 parent isolates underwent long-read sequencing only since short reads for these isolates were available through NCBI SRA (SAMN16887363 and SAMN16887368). Sequencing and assembly approaches are detailed in a companion article (26).

To characterize sequence variations, short-read sequences from the HFIM mutants were aligned to the annotated AR-1049 and AR-1054 complete parent genomes using BWA v0.7.15 (27). Mutations were identified from alignments using *breseq* and the bcftools filter analysis pipelines (28). The *bla*_KPC_ copy number was estimated by dividing the average coverage of the *bla*_KPC_ gene in the alignments to the average coverage across the whole genome. Sequence variations were also assessed via complete genome alignments using progressiveMauve (build date 25 February 2015) (29). Plasmid rearrangements were visualized using Easyfig (v2.2.5; minimum BLAST length 12,000 bp and identity of 0.95) (30). Resistance genes were identified using ResFinder v4.7.2 and AMRFinderPlus v4.0.3 (31, 32). Replicons were identified using PlasmidFinder using thresholds of 100% identity and sequence coverage (33).

### PK validation using UPLC-MS/MS and data analysis

The PK samples were thawed and diluted fivefold in CAMHB. Calibration standard curves were prepared by spiking 20 mL of serial working standard solutions into 180 mL of CAMHB to achieve the desired concentrations. To each sample and standard, 800 mL of methanol containing 200 ng/mL of the internal standard diclofenac was added. Samples were then centrifuged at 13,000 rpm for 10 min to precipitate proteins. Following centrifugation, 100 mL of the supernatant was diluted in 200 mL of water and injected into the ultra-performance liquid chromatography-tandem mass spectrometry (UPLC-MS/MS) system.

Drug concentrations were determined using an Acquity I-Class UPLC system (Waters, Milford, MA) interfaced with a Triple Quad 6500+ MS/MS system (AB Sciex, Framingham, MA). The UPLC column used was an Acquity UPLC BEH C18 (130 Å, 2.1 × 100 mm, 1.7 µm; Waters, Milford, MA, USA).

For vaborbactam and meropenem, separation was carried out at 45°C with a run time of 3.5 min. The mobile phase consisted of 0.1% formic acid in water (A) and 0.1% formic acid in acetonitrile (B), at a flow rate of 0.4 mL/min. The gradient was as follows: 5% B (0–1.0 min), 5%–60% B (1.0–1.2 min), 60%–80% B (1.2–2.4 min), 80%–5% B (2.4–2.5 min), and 5% B (2.5–3.5 min). The injection volume was 5.0 mL. The MS/MS system was operated in positive mode for meropenem and negative mode for vaborbactam using the turbo spray IonDrive. Source/gas parameters were: curtain gas, 30 psi; collision gas, 9 psi; ion spray voltage, +5,500 V/−4,500 V; source temperature, 550°C; ion source gas 1 and 2, both at 35 psi. The optimized multiple reaction monitoring (MRM) transitions were: m/z 384.2→141.10 for meropenem, m/z 296.0→215.1 for diclofenac, in positive mode; m/z 296.0→234.0 for vaborbactam, m/z 294.0→214.1 for diclofenac, in negative mode. Dwell time was 30 ms for all compounds.

For gentamicin and plazomicin, separation was performed using the same UPLC column at 40°C with a 3.5 min run time. The mobile phases consisted of 10 mM ammonium acetate with 0.14% trifluoroacetic acid in water (A) and 0.1% formic acid in acetonitrile (B), at a flow rate of 0.4 mL/min. The gradient was: 5% B (0–0.3 min), 5%–20% B (0.3–1.0 min), 20%–40% B (1.0–1.5 min), 40%–90% B (1.5–2.0 min), 90% B (2.0–2.6 min), 90%–5% B (2.6–2.8 min), and 5% B (2.8–3.5 min). The injection volume was 10 mL. The MS/MS system was operated in positive ion mode. Source/gas parameters were: curtain gas, 25 psi; collision gas, 9 psi; ion spray voltage, +5,500 V; source temperature, 550°C; ion source gas 1 and 2, both at 30 psi. MRM transitions were: m/z 593.4→434.3 for plazomicin, m/z 478.5→157.2 for gentamicin, and m/z 296.0→215.1 for diclofenac. Dwell time was 20 ms for all compounds.

Peak integration and data analysis were performed in Analyst software (AB Sciex, Framingham, MA). Samples were quantified against calibration curves generated in each analytical batch. Assay performance was validated based on established acceptance criteria (34). Assay precision/accuracy (mean %) were as follows: 21.9%/0.5% at 0.1 mg/L (lower limit of quantification [LLOQ]), 8.1%/7.4% at 1.0 mg/L, 9.7%/−10.7% at 10 mg/L, and 8.6%/13.1% at 50 mg/L for vaborbactam; 7.7%/−2.9% at 0.3 mg/L (LLOQ), 5.6%/7.2% at 1.0 mg/L, 11.6%/−2.4% at 5 mg/L, and 10.8%/−1.8% at 30 mg/L for meropenem; 11.9%/0.8% at 0.1 mg/L (LLOQ), −8.8%/−4.3% at 1.0 mg/L, 1.2%/1.9% at 10 mg/L, and 9.2%/−0.9% at 100 mg/L of gentamicin; 16.4%/1.8% at 0.01 mg/L (LLOQ), 8.2%/2.2% at 0.1 mg/L, 1.5%/2.6% at 10 mg/L, and 2.7%/−1.3% at 100 mg/L of plazomicin.

Observed antibiotic concentrations measured by UPLC-MS/MS from the central reservoir following doses at 24 h were used for a noncompartmental analysis using PKanalix (Simulations Plus, Inc.). Actual PK parameters in the system were determined (*f*AUC_24_, *fC*_max_, CL, and *T*_1/2_) and compared to initial predicted values (Table 2).

## RESULTS

### Lower mutation frequency rates did not correlate with better meropenem/vaborbactam activity in time-kill assays

**Fig 1:**
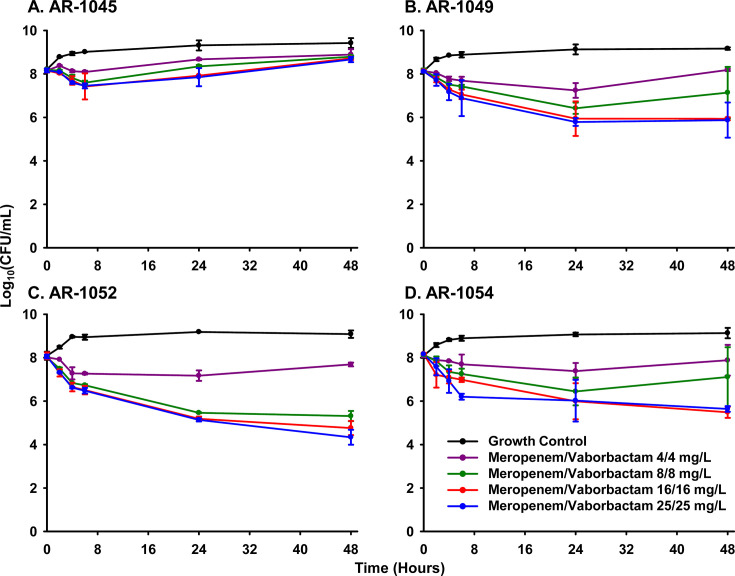
Comparison of the meropenem/vaborbactam activity in time-kill assays against four KPC-Kp isolates with the same meropenem/vaborbactam MICs (2/8 mg/L) but with differing mutation frequencies. The mutation frequencies were <2 × 10^−9^ (**A and C**) or 2.65–3.19 × 10^−7^ (**B and D**). The data are presented as the mean ± SD of the log_10_ CFU/mL. Bactericidal activity: ≥3-log_10_ CFU/mL reduction from baseline.

Colonies capable of growing on meropenem/vaborbactam-containing agar (8/8 mg/L) were detected for 2 of 16 isolates (12.5%; Table 1). These 2 isolates (AR-1049 and AR-1054) each had meropenem/vaborbactam MICs of 2/8 mg/L and mutation frequencies of 2.65–3.19 × 10^−7^, whereas the other 14 isolates had mutation frequencies of <10^−9^. The four isolates with meropenem/vaborbactam MICs of 2/8 mg/L were evaluated in time-kill assays to determine if activity corresponded to the mutation frequency. There was no clear pattern observed to correlate mutation frequency and activity or regrowth in time-kill assays (Fig. 1). There was a trend toward regrowth with meropenem/vaborbactam 8/8 mg/L against the higher mutation frequency isolates, though the difference between viable counts at 24 and 48 h was not significant. Among the four isolates, the least amount of killing by meropenem/vaborbactam in time-kill assays was observed against AR-1045, which had a low mutation frequency. For this isolate, some regrowth occurred from 6 to 48 h for all tested drug concentrations.

### Meropenem/vaborbactam alone and in combination with aminoglycosides overcomes KPC-Kp isolates with lower MICs

**Fig 2:**
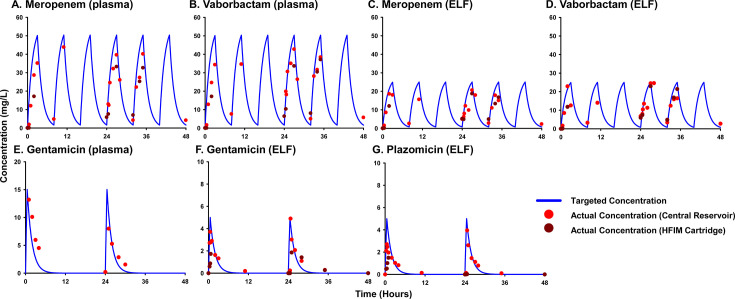
Observed versus targeted PK profiles for meropenem (**A, C**), vaborbactam (**B, D**), gentamicin (**E, F**), and plazomicin (**G**) in the HFIM. Observed data were obtained in drug-only experimental runs, except for the plasma data for gentamicin, which was based on samples collected during an experiment with bacteria.

**Fig 3:**
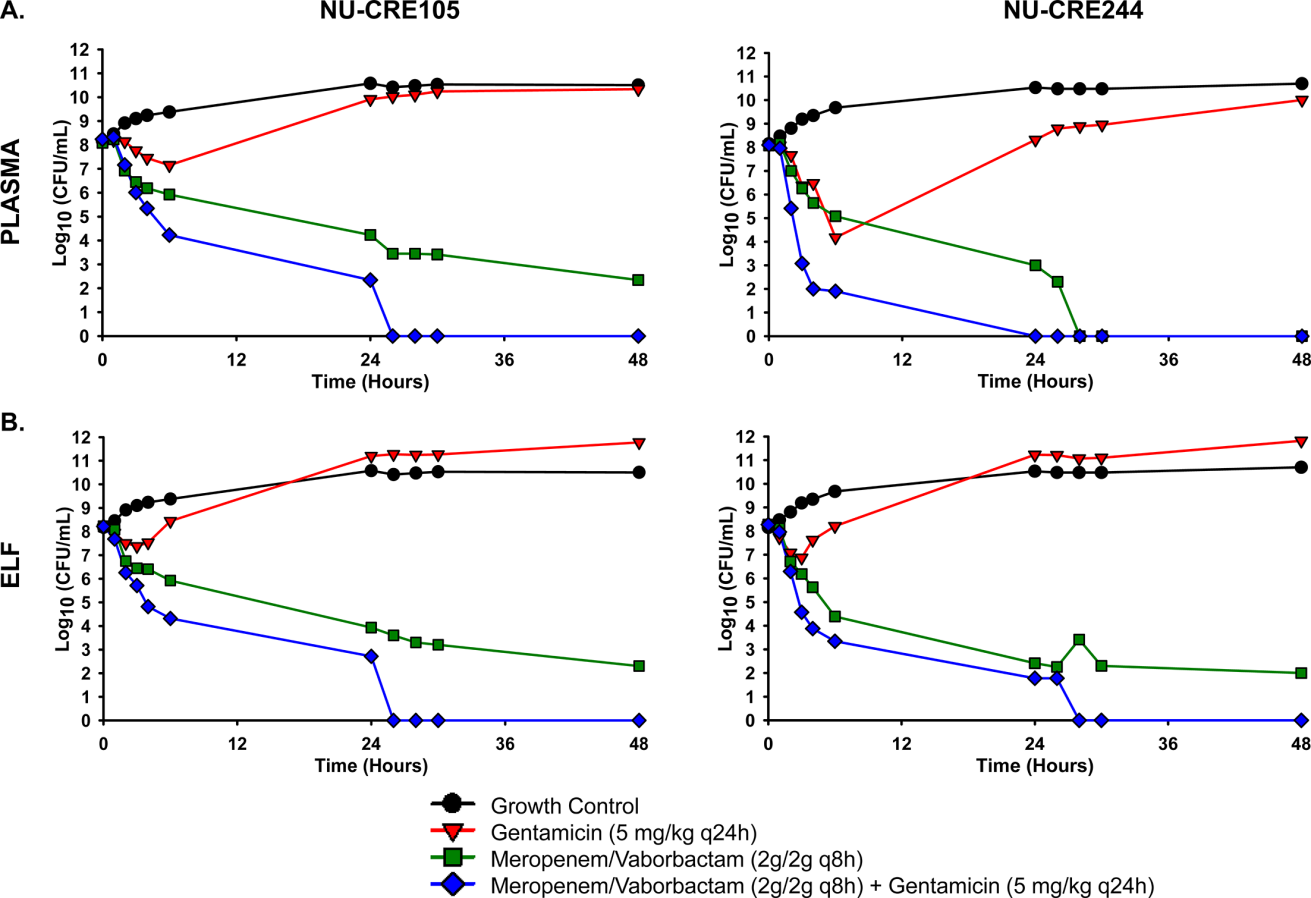
Pharmacodynamic activity of meropenem/vaborbactam alone and in combination with gentamicin against KPC-Kp NU-CRE105 and NU-CRE244 in the HFIM. Antibiotic exposures simulated PKs in the plasma (**A**) and ELF (**B**) following human doses of meropenem/vaborbactam and gentamicin. Bactericidal activity: ≥3-log_10_ CFU/mL reduction from baseline.

The pharmacodynamic activities of meropenem/vaborbactam, gentamicin, and their combination were evaluated in the HFIM against KPC-Kp with meropenem/vaborbactam MICs of 0.125/8–0.25/8 mg/L over 2 days. Antibiotics were tested using exposures that mimicked PK profiles in the plasma and ELF following clinically used doses. Observed antibiotic concentrations in the HFIM adequately matched predicted concentration profiles (Table 2, Fig. 2). Across all of the doses with available peak concentrations between 0 and 48 h, the mean *fC*_max_ ± SD (mg/L) were 39.9 ± 0.4 (meropenem; plasma), 17.8 ± 4.0 (meropenem; ELF), 40.6 ± 5.5 (vaborbactam; plasma), 20.5 ± 2.1 (vaborbactam; ELF), 10.6 ± 3.7 (gentamicin; plasma), 4.3 ± 0.8 (gentamicin; ELF), and 3.3 ± 0.9 (plazomicin; ELF). When testing simulated plasma PK (Fig. 3A), gentamicin caused initial killing of 0.97 and 3.94 log_10_(CFU/mL) for NU-CRE105 and NU-CRE244, respectively. However, regrowth for both isolates occurred despite achieving an *fC*_max_/MIC of 30.

The meropenem %*fT* > MIC, calculated using the meropenem/vaborbactam MIC, was 100% for plasma and ELF regimens against both isolates. Meropenem/vaborbactam plasma exposures caused a steady decline in viable counts for both isolates throughout the experiment, with reductions at 48 h of >5 log_10_ CFU/mL (Fig. 3A). Meropenem/vaborbactam plus gentamicin combinations were synergistic from 6 to 48 h for NU-CRE105 and from 3 to 26 h for NU-CRE244 and achieved undetectable counts by 26 h for both strains.

Comparable pharmacodynamic activity was achieved for PK profiles mimicking ELF exposures (Fig. 3B). The activity of gentamicin was slightly diminished for ELF exposures, with regrowth beginning at least 3 h sooner than was observed following simulated plasma exposures. ELF exposure of meropenem/vaborbactam caused similar killing of NU-CRE105 compared to the plasma exposure. In contrast, there was a 2-log_10_ CFU/mL decrease in the meropenem/vaborbactam activity when using ELF exposures against NU-CRE244 between 28 and 48 h.

### Combinations suppressed meropenem/vaborbactam-resistance emergence in KPC-Kp isolates with higher MICs

**Fig 4:**
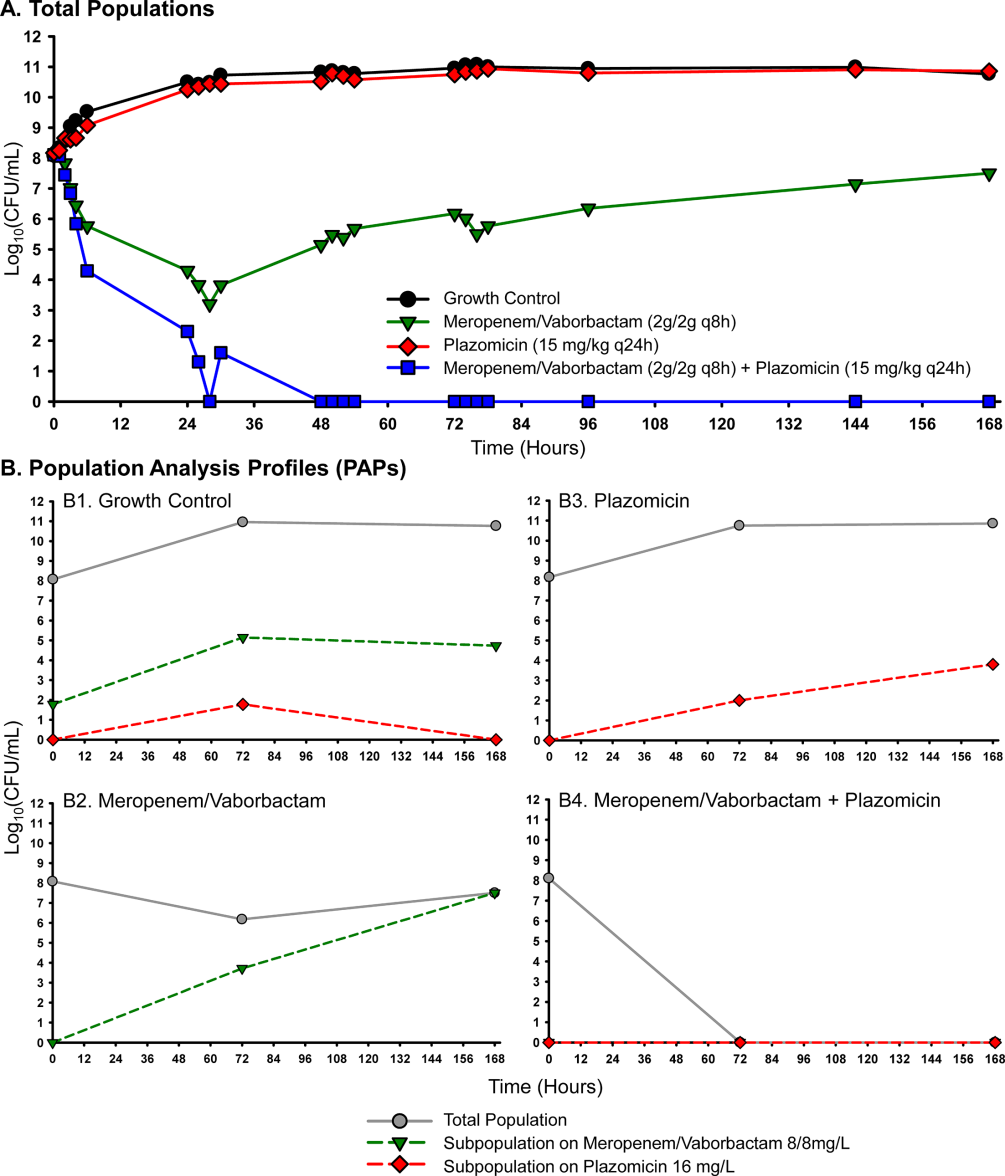
Pharmacodynamic activity of meropenem/vaborbactam alone and in combination with plazomicin against KPC-Kp AR-1049 in the HFIM. Antibiotic exposures simulated PKs in the ELF following human doses of each antibiotic. Viable colonies were tracked for the total population on MHA (**A**) and for resistant subpopulations (**B**) over 168 h. Bactericidal activity: ≥3-log_10_ CFU/mL reduction from baseline.

**Fig 5:**
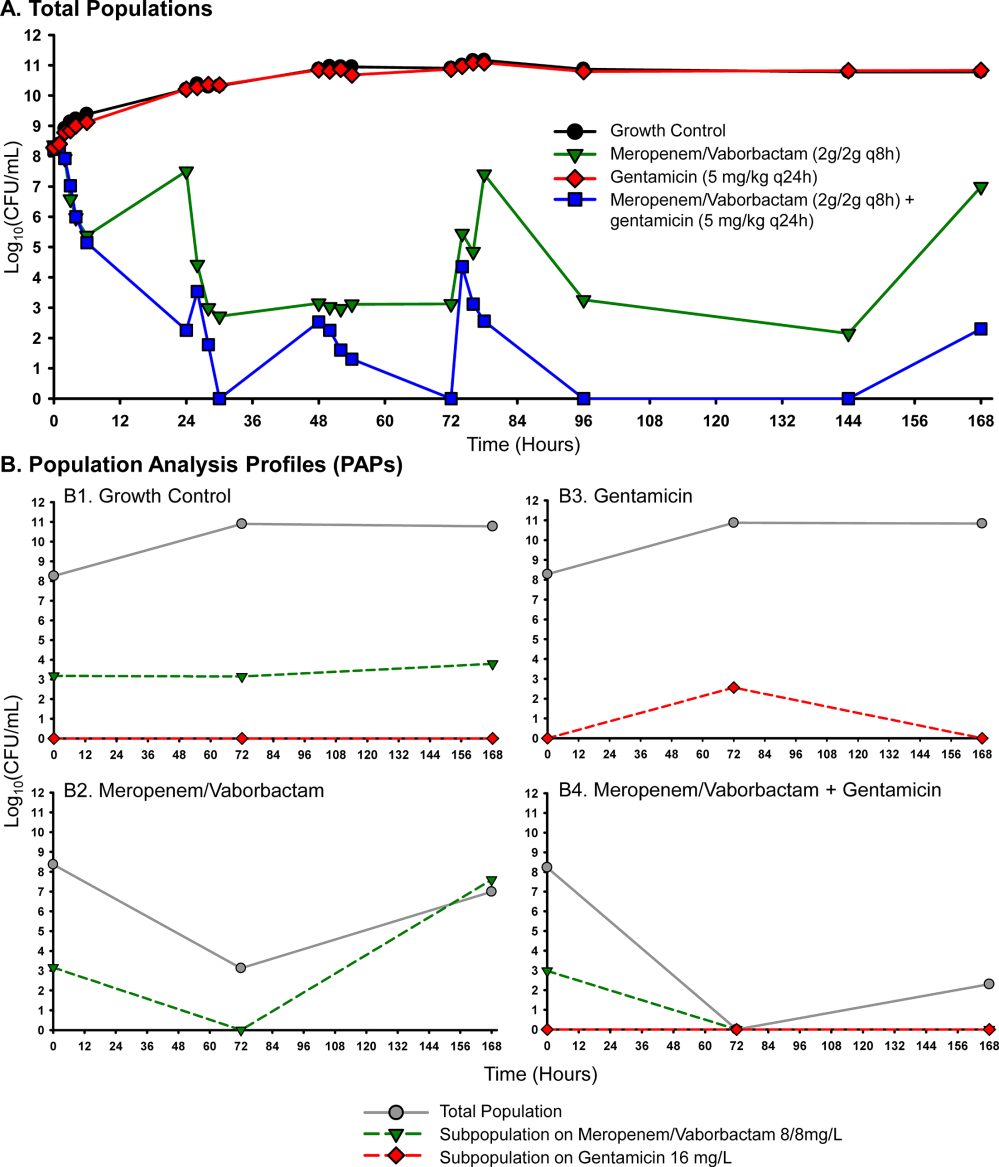
Pharmacodynamic activity of meropenem/vaborbactam alone and in combination with gentamicin against KPC-Kp AR-1054 in the HFIM. Antibiotic exposures simulated PKs in the ELF following human doses of each antibiotic. Viable colonies were tracked for the total population on MHA (**A**) and for resistant subpopulations (**B**) over 168 h. Bactericidal activity: ≥3-log_10_ CFU/mL reduction from baseline.

Two isolates with meropenem/vaborbactam MICs (2/8 mg/L) close to the susceptibility breakpoint (≤4/8 mg/L) were tested against meropenem/vaborbactam alone and with an aminoglycoside (gentamicin or plazomicin) at simulated ELF PK in the HFIM over 7 days. Against each KPC-Kp isolate, aminoglycoside monotherapies failed to inhibit the growth, and some resistant subpopulations emerged during the course of treatment (Fig. 4 and 5). Meropenem/vaborbactam alone was initially bactericidal against each isolate, but resistance began to emerge over time, and nearly the entire population at 168 h was capable of growing on agar plates embedded with meropenem/vaborbactam 8/8 mg/L (Fig. 4B2 and 5B2). Importantly, the meropenem/vaborbactam plus aminoglycoside combinations were capable of reducing regrowth and suppressing the emergence of meropenem/vaborbactam resistance. These combinations led to 7.5 and 4.5 log_10_CFU/mL greater reductions at 168 h compared to meropenem/vaborbactam alone for isolates AR-1049 and AR-1054, respectively.

**Fig 6:**
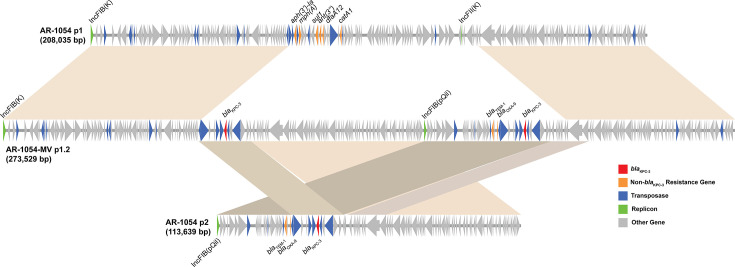
A fusion between two plasmids occurred in AR-1054 following exposure to meropenem/vaborbactam in the HFIM and emergence of resistance (AR-1054-MV). Plasmids p1 (top) and p2 (bottom) from the parental strain fused to form a single 273,529 bp plasmid p1.2 (middle), which includes two copies of *bla*_KPC_ (red).

Colonies growing on meropenem/vaborbactam-containing plates at 168 h were also confirmed by BMD to be resistant to meropenem/vaborbactam with MICs >32/8 mg/L for both AR-1049 and AR-1054. WGS was performed on bacteria extracted after 168 h of exposure to meropenem/vaborbactam (named “AR-1049-MV” and “AR-1054-MV”) or no antibiotics (named “AR-1049-GC” and “AR-1054-GC”) in the HFIM. A detailed description of the complete genome assemblies is provided in a companion article (26). The emergence of meropenem/vaborbactam resistance in AR-1054-MV culminated with the fusion between two parental strain plasmids (p1: 208,035 bp and p2: 113,639 bp) to form a single plasmid (p1.2: 273,529 bp) that consists of a 71.2 kb deletion of plasmid p1 into which a partial duplication of plasmid p2 is inserted, resulting in plasmid p1.2 containing two copies of *bla*_KPC_ (Fig. 6). Based on relative read coverages, there was an apparent 3× increase in the *bla*_KPC_ copy number for AR-1054-MV compared to AR-1054-GC (Table S1). An insertion of an IS5-like element in the promoter of *ompK36* (−8 bp upstream), as well as additional nonsynonymous mutations in an acridine efflux pump and hypothetical protein, was also identified (Table S2). The putative cause of meropenem/vaborbactam resistance in AR-1049-MV was less obvious but may be in part related to a 3-nucleotide deletion in a lysyl-tRNA synthetase gene paired with a duplication of lysyl-tRNA (Table S2). Rearrangements occurred on the *bla*_KPC_-harboring plasmid, but they were located >30 kb away from the *bla*_KPC_ gene, and there was no apparent copy number change (Table S1). Furthermore, an inversion in the same region was detected in the absence of antibiotic exposure (AR-1049-GC; Fig. S1). Together, these findings suggest the cause of meropenem/vaborbactam resistance is not related to *bla*_KPC_ for AR-1049-MV, but that the *bla*_KPC_-harboring plasmid may contain a region prone to rearrangements irrespective of antibiotic selection. Other mutations were identified in nutrient transporters (phosphate), metabolic enzymes (fructokinase and acyl-CoA dehydrogenase), and hypothetical proteins.

## DISCUSSION

Meropenem/vaborbactam remains a key therapeutic option for infections caused by KPC-Kp. However, clinical treatment failure and mortality persist in select patient populations, such as those who are critically ill, suggesting that further therapeutic optimization may be beneficial. Here, we show that meropenem/vaborbactam exhibited robust pharmacodynamic activity (bactericidal by 24 h) following simulated plasma and ELF exposure profiles against isolates with MICs of 0.125/8 to 0.25/8 mg/L. In contrast, ELF exposures of meropenem/vaborbactam exhibited poorer activity against isolates with MICs nearer to the susceptibility breakpoint, and resistance emerged during the 7-day treatment in the HFIM. In general, adding an aminoglycoside enhanced the activity of meropenem/vaborbactam; however, the benefit of the combination was more notable in isolates with higher baseline meropenem/vaborbactam MICs, where the combinations were able to suppress the emergence of resistance.

Meropenem/vaborbactam alone has generally demonstrated robust activity against KPC-Kp. The MIC_50/90_ values for meropenem/vaborbactam against KPC-Kp are 0.12 and 1 mg/L, respectively, and ~99% of these isolates are categorized as susceptible (35). Although resistance can emerge during therapy, available data suggest that the frequency of resistance development is lower with meropenem/vaborbactam compared to ceftazidime/avibactam (~5% vs 10%–20%) (7, 9, 36). Prior HFIM studies have demonstrated favorable pharmacodynamic activity of meropenem/vaborbactam against KPC-Kp isolates, though these experiments were only conducted for ≤32 h (20, 37). In one of these previous studies, Sabet et al. simulated human PK of multiple dosing regimens and showed that KPC-Kp isolates with meropenem/vaborbactam MICs of ≤4/8 mg/L could be killed by ≥5 log_10_CFU/mL within 32 h with the 2 g/2 g dose administered every 8 h. In the present study, meropenem/vaborbactam simulating a human dose of 2 g/2 g administered every 8 h produced robust killing of two isolates with lower meropenem/vaborbactam MICs (0.125/8 to 0.25/8 mg/L) using plasma PK profiles. Together, these data support that meropenem/vaborbactam is a reliable treatment option for susceptible KPC-Kp when antibiotic concentrations meet or exceed those achieved in the bloodstream.

Although meropenem/vaborbactam is not formally approved by the FDA for the treatment of bacterial pneumonia, it is sometimes used off-label to treat such infections when KPC-Kp is the causative pathogen (9). To inform its potential efficacy in this context, we evaluated the activity of meropenem/vaborbactam at regimens simulating exposures in ELF over a longer time period (i.e., 7 days) than has been previously studied in HFIM. In our study, considerable resistance emerged by day 7 in two KPC-Kp isolates with meropenem/vaborbactam MICs of 2/8 mg/L. Although Sabet et al. did not directly simulate ELF exposures in their HFIM study, they did mimic exposures following a 1 g/1 g dose, which employed comparable PK parameters to the ELF regimens that we tested here (20). Similar to our study, they found that the 1 g/1 g simulated dose led to a bifurcation at an MIC of 0.5/8 mg/L; isolates with MICs ≤0.5/8 mg/L were adequately killed by meropenem/vaborbactam, but regrowth and resistance emergence of the KPC-Kp isolate with an MIC of 1/8 mg/L were detected. By doubling the vaborbactam dose (while keeping a fixed meropenem dose), resistance emergence in that same KPC-Kp isolate could be suppressed, suggesting that the exposure of the β-lactamase inhibitor is of significance. The meropenem exposures in ELF following a 2 g dose administered every 8 h as a 3 h prolonged infusion should exceed the required meropenem PK/PD target (45% *fT* > MIC associated with a 2-log_10_ CFU/mL reduction in murine thigh infection models) for isolates with meropenem/vaborbactam MIC values up to 8/8 mg/L in most patients (38). However, vaborbactam exposures in the ELF may only enable targeting isolates with MICs up to 0.5/8 or 1/8 mg/L when using the *f*AUC/MIC target of 220 (associated with a 2-log_10_ CFU/mL reduction in the murine thigh infection model) (37). An *f*AUC/MIC target of 38, which is associated with a 1-log_10_ CFU/mL reduction in murine thigh infection models, may be achievable for most patients that have isolates with MICs up to 8/8 mg/L. Taken together, these data suggest that a meropenem/vaborbactam dose of 2 g/2 g every 8 h as a 3 h prolonged infusion may have some vulnerability when used to treat pneumonia with susceptible isolates that have MICs of 1/8 to 4/8 mg/L. Clinical studies that assess meropenem/vaborbactam for the treatment of pneumonia are required to validate these findings.

Several studies have evaluated the activity of meropenem/vaborbactam for the treatment of pneumonia. In a murine lung infection model, meropenem/vaborbactam achieved >1.5 log_10_CFU/mL reductions at 24 h against two KPC-Kp isolates with MICs of ≤0.06/8 and 0.5/8 mg/L (39). Of note, isolates with higher meropenem/vaborbactam MICs were not tested in this model. Clinically, meropenem/vaborbactam has demonstrated similar 30-day mortality rates for the treatment of pneumonia when compared to bloodstream infections, suggesting that the lower antibiotic exposures in the ELF may not necessarily translate to worse patient outcomes (9). Nonetheless, these results are difficult to interpret due to confounding factors such as differences in baseline comorbidities, as well as the severity and type of infection. Moreover, MIC data are not routinely reported, and antibiotic combinations are used inconsistently. In the TANGO II randomized trial, only four patients with pneumonia were included in the primary analysis population who received meropenem/vaborbactam, and MICs were not reported for these cases (6). Collectively, these data suggest that meropenem/vaborbactam monotherapy is a reliable treatment option for KPC-Kp infections but that some caution may be warranted for the treatment of KPC-Kp pneumonia when the MIC is ≥1/8 mg/L.

Elevated meropenem/vaborbactam MICs in KPC-Kp isolates are often attributed to increased expression of *bla*_KPC_ and mutations in outer membrane porins (12). Despite being susceptible to meropenem/vaborbactam, AR-1049 and AR-1054 had high baseline MICs, likely related at least in part to mutations in *ompK35/36*. In both AR-1049 and AR-1054, *ompK35* was disrupted by a frameshift mutation at nucleotide position 242, and the *ompK36* gene in AR-1049 was disrupted by a frameshift mutation at position 166. The *ompK36* gene in AR-1054 had a two-amino acid insertion (D135DGD) at baseline, which can increase carbapenem MICs (12, 40). Meropenem/vaborbactam resistance emerged in each of these isolates following treatment in the HFIM (MICs >32/8 mg/L). The cause of this MIC shift in AR-1054-MV is likely related to the duplication of *bla*_KPC_ due to the formation of a new fusion plasmid in addition to the insertion of a transposon upstream of *ompK36*, similar to what has been previously described. (12) In AR-1049-MV, the putative cause of the emergence of meropenem/vaborbactam resistance is not as clear, though it is possibly related to a mutation in the lysyl-tRNA synthetase gene, as has been previously identified in carbapenem-resistant *Pseudomonas aeruginosa* isolates (41). However, mutations in the lysyl-tRNA synthetase gene may be more likely to cause antibiotic tolerance than a direct MIC increase (42). Interestingly, there was also a duplication of the lysyl-tRNA gene in this isolate, which might function together with the mutation in the lysyl-tRNA synthetase to alter bacterial translation and stress response. Several other mutations were identified in nutrient transporters, metabolic enzymes, and hypothetical proteins, suggesting that resistance may have been driven by a global physiology shift rather than single changes. Additional functional experiments will be required to define the specific contributions of each mutation.

One strategy that may enhance the activity of meropenem/vaborbactam against KPC-Kp and mitigate the risk of resistance emergence is combination therapy with an aminoglycoside. We previously found that aminoglycosides displayed synergy with ceftazidime/avibactam against KPC-Kp in both the HFIM (17) and a subanalysis of a retrospective cohort study (43). In the present study, which was the first to evaluate combinations between meropenem/vaborbactam and aminoglycosides, the combination was able to suppress meropenem/vaborbactam resistance from developing. Aminoglycosides were selected based on their activity against each isolate. For example, plazomicin was studied against AR-1049 since this isolate harbored resistance genes for amikacin, gentamicin, and tobramycin (*aac (3)-IV* and *aac(6')-Ib*). All other isolates remained susceptible to gentamicin. To implement a similar combination strategy in patients, phenotypic susceptibility testing would be required to select the aminoglycoside. Our group and others have also shown that aminoglycoside activity can be adequately predicted by the isolate’s resistance genes (44–47). Coupled with advances in rapid diagnostics, this molecular information is becoming increasingly accessible to clinicians. Several potential explanations may explain the synergy observed between these antibiotics, as hypothesized previously (48). One possibility is that the aminoglycosides may kill pre-existing meropenem/vaborbactam-resistant subpopulations and may also prevent new ones from emerging (49). A second possibility is that aberrant proteins induced by the presence of the aminoglycoside may lead to alterations in the structure of the outer membrane or expression of β-lactamases that subsequently enhance the activity of meropenem/vaborbactam (50). Additional studies are ongoing to help elucidate the mechanism(s) of synergy for this combination. Future studies that seek to identify other synergistic partners for KPC-Kp-active β-lactam/β-lactamase inhibitors, such as meropenem/vaborbactam.

There are a few key limitations to the present study. First, the HFIM experiments were conducted in singlicate, and therefore, statistical comparisons were not possible. Thus, small log_10_ CFU/mL differences between regimens should be cautiously interpreted. However, it should be noted that KPC-Kp isolates with similar meropenem/vaborbactam MICs responded similarly to treatment in the HFIM (e.g., NU-CRE105 and NU-CRE244), enhancing confidence in the reproducibility of these data. Second, only active aminoglycosides were included in the combinations. Additional studies are needed to determine, for example, if gentamicin is useful in combination with meropenem/vaborbactam against gentamicin-resistant KPC-Kp. Third, the KPC-Kp isolates with lower meropenem/vaborbactam MICs were tested in shorter duration HFIM studies (48 vs 168 h) so we cannot rule out the possibility that regrowth was unique to isolates with MICs near to the breakpoint. However, during the overlapping 48 h, meropenem/vaborbactam produced consistent killing against the isolates with lower MICs, whereas periods of regrowth were noted for isolates with higher MICs.

In conclusion, meropenem/vaborbactam regimens simulating human plasma and ELF exposure profiles displayed adequate pharmacodynamic activity when tested against highly susceptible KPC-Kp. However, ELF exposures of meropenem/vaborbactam resulted in the emergence of resistance for KPC-Kp with MICs of 2/8 mg/L. Combinations with aminoglycosides were able to considerably enhance bacterial killing by meropenem/vaborbactam against all KPC-Kp strains and suppressed the emergence of resistance. These data suggest that meropenem/vaborbactam is generally an active agent for susceptible KPC-Kp, but some caution may be warranted when using it alone to treat KPC-Kp pneumonia with MICs near the susceptibility breakpoint. Moreover, this study supports consideration of aminoglycosides as potential partner agents to extend the utility of meropenem/vaborbactam in the management of difficult-to-treat KPC-Kp infections. Future clinical studies are required to validate these findings.

## Data Availability

Sequence data are deposited under NCBI BioProject accession no. PRJNA1374223.

## SUPPLEMENTAL MATERIAL

Supplemental material aac.01365-25-s0001.docx Fig. S1; Tables S1 and S2.

ASM does not own the copyrights to Supplemental Material that may be linked to, or accessed through, an article. The authors have granted ASM a non-exclusive, world-wide license to publish the Supplemental Material files. Please contact the corresponding author directly for reuse.
