# Supplementary material for: Aminoglycosides enhance meropenem/vaborbactam activity against KPC-producing Klebsiella pneumoniae in the hollow fiber infection model
Source: Antimicrob Agents Chemother. 2026 Feb 19;70(4):e01365-25. doi: 10.1128/aac.01365-25 (PMC13041348; doi:10.1128/aac.01365-25)
Supplement: Supplemental material — Fig. S1; Tables S1 and S2. [file aac.01365-25-s0001.docx]

**SUPPLEMENT**

**Aminoglycosides Enhance Meropenem/Vaborbactam Activity Against KPC-Producing *Klebsiella pneumoniae* in the Hollow Fiber Infection Model**

Nidhi Singh^1^, Christin M.L. Jogan^1^, Yanan Zang^2^, Xindi Shan^2,3^, Yinzhi Lang^2,3^, Arunkumar Karunanidhi^1^, Jackson V. Watkins^1^, Brooke N. Curry^1^, Pranita D. Tamma^4^, Sophie H. Nozick^5^, Egon A. Ozer^6^, Alan R. Hauser^5^, Jürgen B. Bulitta^2,3^, Zackery P. Bulman^1^

^1^Department of Pharmacy Practice, Retzky College of Pharmacy, University of Illinois Chicago, Chicago, IL, USA

^2^Department of Pharmacotherapy and Translational Research, College of Pharmacy, University of Florida, Orlando, Florida, USA

^3^Department of Pharmacy and Pharmaceutical Sciences, St Jude Children's Research Hospital, Memphis, Tennessee, USA

^4^Department of Pediatrics, Division of Infectious Diseases, Johns Hopkins University School of Medicine Baltimore, Maryland, USA

^5^Department of Microbiology-Immunology, Northwestern University Feinberg School of Medicine, Chicago, Illinois, USA

^6^Division of Infectious Diseases, Department of Medicine, Northwestern University Medical School, Chicago, Illinois, USA

**Table S1.** Average short-read coverage of the *bla*_KPC_ genes in isolates AR1049 and AR1054 relative to the average coverage of the whole genome following meropenem/vaborbactam treatment-emergent resistance in the hollow fiber infection model provides an estimate of the relative *bla*_KPC_ copy number.

| **Isolate** | **HFIM Treatment Regimen (Sample Time)** | **Estimated *bla*_KPC_ Copy Number** |
| --- | --- | --- |
| AR1049 | Growth Control (168 h) | 5.02 |
|  | Meropenem/Vaborbactam (168 h) | 4.33 |
| AR1054 | Growth Control (168 h) | 2.53 |
|  | Meropenem/Vaborbactam (168 h) | 7.33 |

**Table S2.** Single nucleotide polymorphisms, gene duplications, truncations, insertions, and transversions identified in isolates AR1049 and AR1054 after growth in the hollow fiber infection model for 168 h in the absence of antibiotic (growth control) or in the presence of meropenem/vaborbactam.

| **Isolate** | **HFIM Treatment Regimen**  **(Sample Time)** | **Location** | **Gene Product(s)** | **Gene Mutation** | **AA Mutation or Alteration** |
| --- | --- | --- | --- | --- | --- |
| AR1049 | Growth Control (168 h) | Chromosome | LacI transcriptional regulator [KPHS_43290] | ACC -> CCC | T24P |
|  |  | Plasmid 3 | Disruption of one hypothetical protein | Rearrangement between 29701-30830 | Rearrangements and Inversion (see Fig. S1) |
|  | Meropenem/ Vaborbactam (168 h) | Chromosome | glutamine ABC transporter permease [KPHS_16710] | GGC -> GGT | G169G |
|  |  | Chromosome | Lysyl-tRNA synthetase [KPHS_43860] | Δ3 bp | K24del |
|  |  | Chromosome | duplication x 2 of tRNA-Lys [KPHS_t00290] | 3,678,891ins434bp | Duplication |
|  |  | Chromosome | hypothetical protein, transposase, hypothetical protein; truncation of fructokinase [KPHS_13270] | 3,889,945del2,536bp | Deletion |
|  |  | Chromosome | hypothetical protein [KPHS_16060] | 3,660,819insAG | Frameshift |
|  |  | Chromosome | acyl‑CoA dehydrogenase [KPHS_09610] | AUG->AUC | M271I |
|  |  | Chromosome | phosphate transporter permease subunit [KPHS_52990]; transposases x 2 | 5,286,922del781bp | Deletion |
|  |  | Plasmid 1 | hypothetical protein [KPHS_35080]; transposase and small 189bp hypothetical protein | 3,668ins1692bp | Insertion |
|  |  | Plasmid 3 | Disruption of one hypothetical protein | Rearrangement between 29701-30830 | Rearrangements and Inversion (see Fig. S1) |
| **Isolate** | **HFIM Treatment Regimen**  **(Sample Time)** | **Location** | **Gene Product(s)** | **Gene Mutation** | **AA Mutation or Alteration** |
| AR1054 | Growth Control (168 h) | Chromosome | LacI transcriptional regulator [KPHS_43290] | ACC -> CCC | T60P |
|  |  | Chromosome | cysteine/glutathione ABC transporter membrane/ATP-binding protein [KPHS_17970] | CTG -> CAG | L198Q |
|  | Meropenem/ Vaborbactam (168 h) | Chromosome | acridine efflux pump [KPHS_11880] | CAG -> CTG | Q568L |
|  |  | Chromosome | 8bp upstream of OmpK36 porin [KPHS_37010] | IS5-like Transposon insertion at 1,645,497 | Possible disruption of OmpK36 Expression |
|  |  | Chromosome | hypothetical protein [KPHS_35460] | Transposon insertion at 1,828,981 | Insertion |
|  |  | Plasmids 1 and 2 | Multiple | Plasmid Fusion | Many, including the duplication of the KPC gene |


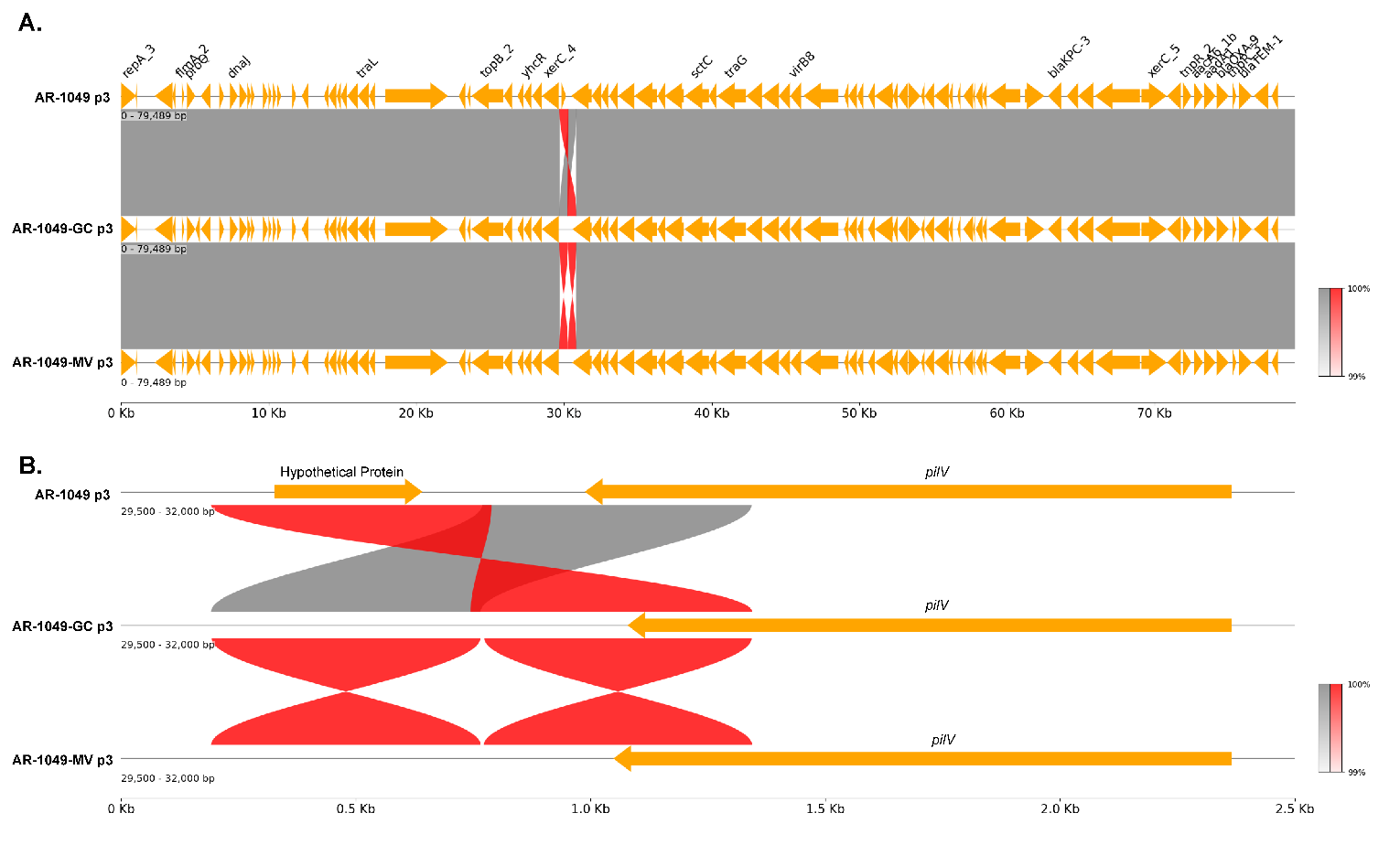
**Figure S1.** Rearrangements occurred in a region of plasmid 3 for AR-1049 following growth in the HFIM in the absence of antibiotics (AR-1049-GC p3) and presence of meropenem/vaborbactam (AR-1049-MV p3) (**A**). Rearrangements and inversions occurred upstream of ­*xerC* (tyrosine recombinase) and affect the position and orientation of a hypothetical protein gene. These rearrangements also impact the sequence of the C-terminal-encoding portion of a *pilV*gene (shufflon system plasmid conjugative transfer pilus tip adhesin PilV) (**B**).
